# Zinc and Zinc Transporters in Dermatology

**DOI:** 10.3390/ijms232416165

**Published:** 2022-12-18

**Authors:** Zubaidah Al-Khafaji, Sofia Brito, Bum-Ho Bin

**Affiliations:** 1 Department of Applied Biotechnology, Ajou University, Suwon 16499, Republic of Korea; 2 Department of Natural Sciences, Ajou University, Suwon 16499, Republic of Korea

**Keywords:** skin, zinc, zinc transporters, AE, SCD-EDS, skin disorders

## Abstract

Zinc is an important trace mineral in the human body and a daily intake of zinc is required to maintain a healthy status. Over the past decades, zinc has been used in formulating topical and systemic therapies for various skin disorders owing to its wound healing and antimicrobial properties. Zinc transporters play a major role in maintaining the integrity of the integumentary system by controlling zinc homeostasis within dermal layers. Mutations and abnormal function of zinc-transporting proteins can lead to disease development, such as spondylocheirodysplastic Ehlers–Danlos syndrome (SCD-EDS) and acrodermatitis enteropathica (AE) which can be fatal if left untreated. This review discusses the layers of the skin, the importance of zinc and zinc transporters in each layer, and the various skin disorders caused by zinc deficiency, in addition to zinc-containing compounds used for treating different skin disorders and skin protection.

## 1. Zinc Properties

Zinc is the second most abundant trace mineral in the human body [1]. It is a major constituent of every cell and is involved in cellular metabolic activities. The human body cannot make zinc on its own nor store it, therefore, a daily intake of zinc is required to maintain a steady status [2]. Recommended daily intake of zinc for an adult range from 8 to 11mg per day [3]. Zinc plays a major role in DNA synthesis. Zinc-deficient individuals are exposed to DNA damage; leading to impaired growth, delayed sexual maturation, hypogeusia, and hypogonadism [4]. Zinc supports immunity through maintaining Metallothionein (MT) homeostasis of the inflammatory response during aging [5], and insufficient expression of zinc-finger transcription factors in mRNA coding of growth factors leads to impaired wound healing [6]. Zinc also aids in the function of more than 200 enzymes by activating protein metabolism, in addition to protein synthesis [7,8]. As zinc enhances re-epithelialization, it reduces inflammation and bacterial growth [5]. Moreover, zinc supports normal fetal growth and development during pregnancy, childhood, and adolescence [9,10,11].

## 2. The Skin’s Anatomy and Physiology

**Figure 1:**
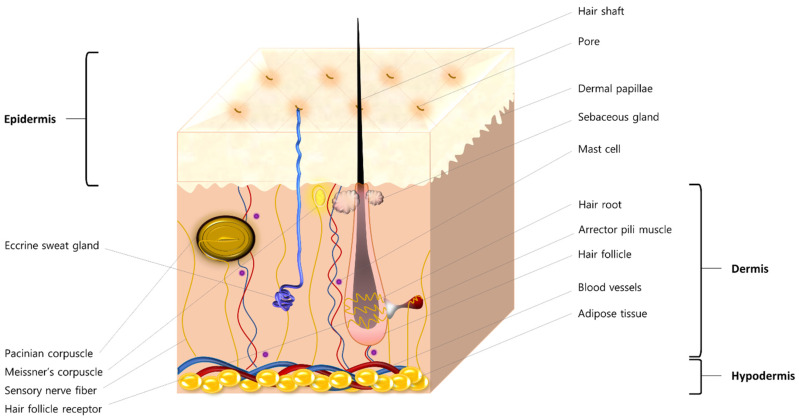
Anatomy of the integumentary system showing different structures within the epidermis, dermis, and hypodermis.

The skin is the body’s largest organ [12], and it is made of fats, protein, water, and minerals. This integumentary system consists of three major layers: the outermost layer or the epidermis, the middle layer or the dermis, and the lowermost layer or the hypodermis. With each layer serving a different purpose, it all pours into protecting the body from microbes and other elements, providing a protective barrier against mechanical, thermal, and physical injury and hazardous substances, preventing loss of moisture, lowering the effects of ultraviolet (UV) rays, in addition to producing vitamin D [13,14] (Figure 1).

### 2.1. The Epidermis

The Epidermis is an avascular layer made of keratinized stratified squamous epithelium comprising four major layers, the stratum corneum, which is the most superficial layer of the epidermis, is a thick layer made up of dead keratinocytes known as corneocytes [15]. Corneocytes protect the skin from injuries, UV light, and microbes [16]. This layer also protects the skin from losing its water content. Deep to the stratum corneum is the stratum lucidum [17], which is a thin layer made of flattened corneocytes that is present on palms and soles [18]. Beneath the stratum lucidum, is the stratum granulosum, which consists of keratinocytes preparing to become flattened corneocytes [19]. Stratum spinosum lays down to the stratum granulosum and contains mature keratinocytes with visible granules that adhere to each other by desmosomes and produce keratin, a protein that helps form hair and nails [20,21]. The deepest layer of the epidermis is the stratum basale or stratum germinativum. This layer contains new keratinocytes in their developing stage, Merkel cells, and stem cells [22]. This layer contains melanocytes that produce melanin responsible for pigmentation.

### 2.2. The Dermis

The dermis is the middle layer of the skin and it is composed of fibrous, filamentous, and amorphous connective tissue [23]. This layer allows stimuli inductions to control entry by the vascular and neural networks, epidermal appendages, fibroblasts, mast cells, macrophages, and other blood cells and it also accommodates sweat glands, sebaceous glands, and hair follicles in addition to allowing substance exchange to both the dermis and the epidermis through the epidermal–dermal junction [24]. The components of the dermal layer, such as collagen, can also vary depending on external stimuli [25]. Collagen is a structural protein that is considered a principal component of the dermal layer with at least 28 genetically different types [26], making up 70% to 80% of the skin’s dry weight [27]. This stress-resistant material is degenerated by spare collagenases [28]. The dermis can be divided into two main regions, the upper papillary layer, and the lower reticular layer [29]. In the papillary and adventitial dermis, Collagen type I fibers are loosely positioned, whereas in the reticular dermis, are present in bulky bundles [29,30]. In the basement membrane, collagen type IV is present, while keratinocytes mainly produce collagen type VII [31].

The dermal vasculature is made of two interrelated plexuses: the superficial subpapillary plexus branches into capillaries that extend to papillae supplying the epidermis, and the deep vascular plexus that is supplied by larger blood vessels extending from the hypodermis [32]. In addition to vascular networks surrounding sweat glands and hair follicles in the dermis, the papillary layer embodies muscle fibers of the arrectores pili that are attached to the hair follicle causing it to be pulled vertically upon contraction resulting in a “goose-bumps” skin condition [33]. The sensation of pain, temperature, and itchiness is attributed to the presence of unmyelinated nerve fibers ending around hair follicles and the papillary layer while mediating touch is regulated by Meissner corpuscles that are mostly present on the palms and soles with more concentration in the fingertips [34,35]. The sensation of pressure is attributed to Pacini corpuscles that are present on the weight-bearing surfaces, genital, nipple, and anogenital reticular dermis [36]. Post-ganglionic adrenergic fibers regulate vasoconstriction through the secretions of apocrine gland secretions and the arrectores pili muscle contractions while the cholinergic fibers regulate eccrine sweat gland secretions [37]. Mast cells are linked to allergic reactions, and they accumulate in large numbers in the papillary dermis and are also found in the subcutaneous fat [38]. Allergens activate mast cells leading to the release of cytokines and chemokines that are responsible for initiating an inflammatory reaction [39].

### 2.3. The Hypodermis

The lowermost layer of the integumentary system, also known as the subcutaneous tissue, is composed of mainly connective tissue and adipose tissue, and it lies between the dermis and the skeletal muscle [40]. The hypodermis contains adipocytes, large blood vessels, nerves, and adipose stromal cells and it is important for thermoregulation, and protection of the underlying structures from traumas, energy reservation, and hormone production (leptin) in addition to supporting keratinocyte and fibroblast proliferation [41]. The deposition of adipocytes in this layer depends on age, hormones, genetic factors, and body region [42,43,44], while the size of adipocytes depend on nutrition [45]. Other components of the hypodermis are macrophages, fibrous bands, collagen, and elastin that connect the subcutaneous layer to the dermis, whereas hair follicle roots can also be found embedded in the hypodermis [46].

## 3. Zinc Distribution in the Human Skin

Zinc is absorbed in the small intestines through a carrier-mediated mechanism [47], and it is distributed to the rest of the body in different amounts. The vast majority goes to the skeletal muscle (60%), followed by the bone (30%) and the skin (5%), the liver (5%), and other organs (2–3%) [48,49,50]. Different illnesses and high phytate-containing foods can inhibit zinc uptake thereby hindering its bioavailability in the body [3]. The skin contains approximately 60 μg/g of zinc in the epidermis and 40 μg/g in the upper dermis [51]. Zinc deficiency can be first exhibited on the skin causing skin diseases such as acrodermatitis enteropathica [3,52].

## 4. Role of Zinc in the Skin

Zinc can bind ~10% of the total proteins identified in the human body, according to bioinformatics research on the human genome. Zinc concentration was found higher in the stratum spinosum than in other epidermal layers [23]. While in the dermis, zinc can be found in higher concentrations in the upper dermis than the lower dermis due to higher mast cell accumulation that is rich in zinc content necessary for cytokine production and FcεRI-dependent degranulation. It was observed that the maintenance of adequate zinc levels within cultured HaCat keratinocytes promotes the survival and proliferation of these cells while zinc deficiency causes activation of a DNA fragment and caspase-3 inducing apoptosis [53]. Extracellular Zn^2+^ is released following skin injury causing activation of the G-protein coupled receptor (GPR39) and zinc-sensing receptor (ZnR) pathway that is expressed in the keratinocytes and other epithelial cells, leading to the repair of the epithelium. Zinc possesses anti-inflammatory properties in suppressing the generation of inflammatory cytokines [54], and the liberation of zinc ions from zinc oxide (ZnO) nanomaterials aids in wound healing [55], therefore, it has been widely utilized in formulating cosmetics and ointments [56,57]. ZnO nanoparticles and Zn^2+^ ions exhibit different spectrums of antimicrobial activities [58,59,60,61,62], and it shows more potent antibacterial properties when combined with chitosan hydrogel, making it a great component for wound dressings [63]. Zinc deficiency impairs the proliferation, differentiation, survival of keratinocytes, and wound healing, in addition to increasing the production of ATP, inflammatory cytokines, and iNOS/NO by keratinocytes in addition to causing a telogen effluvium and an abnormal hair keratinization [49,64]. A less toxic zinc-based metal–organic framework (Zn-BTC), with the ability to slowly release Zn^2+^, was found to exhibit biocompatibility, antibacterial and anti-inflammatory properties. It aids the skin’s wound healing process as Zn-BTC lowers the expression of certain antioxidant genes and enhances the expression of wound healing genes, in addition to, exhibiting better bactericidal effect on different drug-resistant bacteria through reducing 41.4% MRSA and 47.2% Escherichia coli in rats [62]. It was revealed that combining polyethylene glycol (PEG) and ZnO nanoparticles into an FDA-approved bioabsorbable polyester, Poly 4-hydroxybutyrate (P4HB)’s matrix resulted in Zn^2+^ ion release, which allowed for better blood clotting, better bacterial elimination, prevention of bacterial adhesion, and had as well demonstrated excellent hemostatic performance [65]. With the ongoing advancement of nanotechnology in drug delivery, a recent study has confirmed the potential of ZnO nanoparticles in wound healing through the synthesis of LED illuminated curcumin loaded Zinc Oxide (Cu + ZnO NP) nanoparticles that demonstrated an enhanced wound contraction, collagen deposition, angiogenesis, and re-epithelialization, by which in turn, accelerated the overall wound healing process [66]. For skin burns, zinc silicate nanoparticles-based scaffolds were found to enhance Human Umbilical Vein Endothelial Cells (HUVECs) angiogenic activity and Schwann cells’ neurogenic activity, as well as displaying remarkable blood vessel and nerve fiber regeneration abilities, both of which are required for effective skin tissue regeneration, thereafter, it enables a better the healing of innervated and vascularized skin burn wounds [67]. As zinc is required for the integrity of the integumentary tissue, degradation of zinc levels in the skin can lead to a variety of diseases, some are inherited while others are acquired through low dietary intake while other zinc deficiency conditions are linked to intestinal malabsorption [4,68].

## 5. Role of Zinc Transporters in the Skin

**Figure 2:**
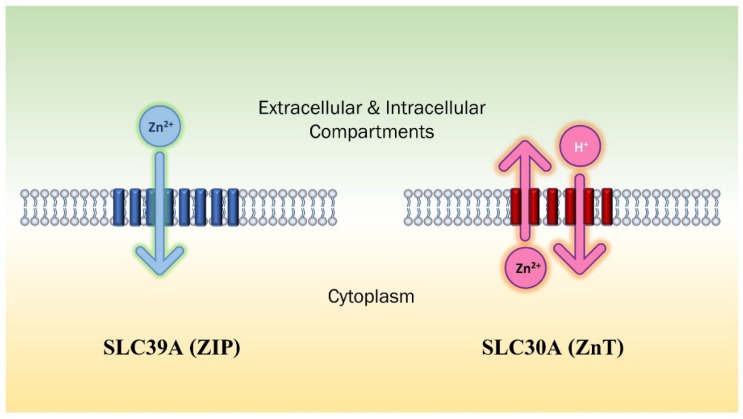
Zinc transporters SLC39A (ZIP) and SLC30A (ZnT) with Zn^2+^ ion transportation direction. ZIP is predicted to have 8 transmembrane domains and ZnT is predicted to have 6.

Zinc transporters are membrane proteins that control the transportation and concentration of zinc both inside the cellular compartments and outside the cells by regulating the influx and efflux of zinc, which is crucial in maintaining homeostasis within the tissues [48]. Primary zinc transporters include the zinc transporter ZnT (SLC30) family and the Zrt/Irt-like protein/solute carrier family ZIP (SLC39) [69,70]. These transporters directly influence zinc availability and pathogenesis. There are ten members of the zinc efflux transporter family (ZnT) and fourteen members of the zinc inflow transporter family (ZIP) that have been identified in mammals [70,71]. According to research, ZnT transporters and their homologs function as Zn^2+^/H^+^ antiporters [72], while ZIP transporters act as a symporter for zinc and other metals/bicarbonates [73,74]. A study suggested that ZIP transporters act as a zinc-selective channel that transports zinc ions into the cytoplasm based on zinc concentration gradients [75]. ZIP family was found to be directly involved in maintaining skin homeostasis [76]. In the epidermis, ZIP1, ZIP2, ZIP4, and ZIP10 are linked to epidermal morphogenesis and abnormalities [23,77,78,79], whereas ZIP7 and ZIP13 are required for normal dermal development and collagen metabolism [80]. Although zinc transporters play a major role in maintaining cellular homeostasis, metallothioneins (MT) accumulating in the epidermal layer, binding heavy metal ions such as zinc are found to be associated with increased zinc concentrations in the tissues which implies the significance of not only ZIP but also MTs in maintaining high concentrations of zinc required for normal proliferation and differentiation of the epidermis [76,81]. Acrodermatitis enteropathica (AE) is a zinc metabolism disorder that can be inherited or acquired, and it is caused by dysfunction of ZIP4 protein leading to impaired zinc absorption [82]. Spondylocheiro dysplastic form of Ehlers–Danlos syndrome (SCD-EDS) is an autosomal-recessive entity that is initiated by mutation of the zinc transporter ZIP13 causing hyperplasia and weakening of the skin and hypermobility in the small joints [83]. Epidermodysplasia verruciformis (EV) is an autosomal-recessive skin disease associated with a high risk of skin cancer [84,85] developing in individuals vulnerable to specific genotypes of human papillomavirus, such as the oncogenic HPV-5. EV patients show mutations in EVER1 or EVER2 genes that form a complex with ZnT1 majorly in the endoplasmic reticulum of keratinocytes leading to an increase in free zinc transportation into the nucleus, and thus, promoting AP-1 activity that causes abnormal replication of EV-related oncogenic HPVs responsible for skin cancer development [49,86]. Poor secretion of zinc into breast milk causes a condition called Transient Neonatal Zinc Deficiency resulting from ZnT2 mutation as ZnT2 transports zinc from the cytoplasm to cytoplasmic secretory vesicles [87,88]. ZIP2 aids in the differentiation of keratinocytes in the epidermis and the knock-down of ZIP2 leads to immortalizing human keratinocytes and inhibiting their differentiation [23] (Figure 2).

## 6. Function of Zinc, and Zinc Transporters in Dermal Skin Cells

### 6.1. Function of Zinc in the Dermal Layer

As the dermal layer consists of connective tissue containing nerve endings, sweat glands, oil glands, and hair follicles, zinc promotes dermal homeostasis through zinc transporters and MT proteins to regulate the levels of zinc across body cells’ phospholipid bilayers [8,76]. Zinc has anti-inflammatory properties and influences main pro-inflammatory signaling pathways as in down-regulating inflammatory cytokines release [89].

### 6.2. Function of Zinc Transporters in the Dermal Layer

There are 14 identified zinc transporters within the ZIP family [70]. Among them, ZIP7 (SLC39A7) and ZIP13 (SLC39A13) zinc-regulating transporters are required for optimal development of the dermal layer [80]. These transporters regulate cytosolic zinc levels by delivering the required zinc quantities for optimal function [90]. ZIP7 transports zinc from the endoplasmic reticulum (ER) stores to the cytoplasm and regulates zinc homeostasis in the Golgi apparatus [91,92]. Whereas ZIP13, a transporter protein required for connective tissue development [93], functions in transporting zinc from the vesicular stores to the ER and other compartments [94]. ZIP13 dysfunction leads to a decrease in zinc levels in the cytoplasm and ER, causing ER dysfunction and stress dysfunction. Thereafter, any abnormalities within these transporters can lead to improper dermal formation, thus causing dermal impairments such as dermal dysplasia [92,95]. Transforming growth factor beta (TGF-β)–SMAD–ZIP13 axis was also found to be necessary for dermal formation [92].

### 6.3. ZIP7 Transporter

ZIP7 is found in the ER promoting zinc homeostasis [80]. However, recent studies suggest that ZIP7 can also be present in the Golgi apparatus [91]. ZIP7 is involved in the formation of the skin’s connective tissue. Unlike other members of the SLC39A family, ZIP7 has a histidine binding region known as N-termini that acts as a zinc ligand maintaining homeostasis within the cell’s ER lumen where ZIP7 resides along with labile zinc [96]. ZIP7-knockout mice showed reduced dermal and hypodermal thickness, indicating ZIP7 is required for connective tissue development [92]. ZIP7-knockout leads to decreased cell density, thereby, thinner connective tissue indicating that ZIP7 is crucial for human mesenchymal stem cell (hMSC) proliferation [92]. ZIP7 is more predominant in the hMSCs than ZIP13 and is found to be essential in fibrogenic and osteogenic development suggesting ZIP7 is required for preventing ER stress and thus, preservation of hMSCs [80].

Contrary to ZIP13, the knockdown of ZIP7 in cells exhibits enhanced ER stress, accompanied by higher zinc concentrations and aggregation of protein disulfide isomerase (PDI) leading to unfolded protein response, suppressing cell growth and eventually, apoptosis [76].

In pre-B and immature B cells, ZIP7 is necessary for promoting gene transcription affecting the normal transition from late pre-B to immature B cells. In activated human primary B cells, about 50% ZIP7-containing compartments were found within 1 µm of the plasma membrane while more widespread distribution was observed in the cytoplasm of considerably bigger HEK293T cells. This finding indicates that alterations in local or dynamic ZIP7 dissemination of Zn^2+^ can vary by cell type [97]. ZIP7 deficiency stimulates the death of normally developing B cells, disrupting its differentiation process as it fundamentally modifies the gene expression program according to RNA sequencing. The decreased cytoplasmic Zn^2+^ associated with ZIP7 deficiency is expected to result in pathologically elevated phosphatase activity and, as a result, contribute to impaired pre-BCR- and BCR-dependent signaling at the positive selection checkpoints. By employing flow cytometry and a cell-permeable alkaline phosphatase substrate, it was discovered that ZIP7 had greater constitutive phosphatase activity than WT pre-B and immature B cells. 

### 6.4. ZIP13 Transporter

ZIP13 regulates zinc homeostasis in the Golgi apparatus [80]. ZIP13 is a homo-dimerized zinc transporter containing eight transmembrane domains with N and C termini in the hydrophilic region [95]. The expression of Drosophila ZIP13 (dZIP13) in Saccharomyces cerevisiae revealed that it is mainly regulated by iron abundance in protein level with a slight iron response within mRNA as the presence of iron in excess amounts activates iron binding affinity boosting its efflux and preventing dZIP13 from deterioration [98].

It was found that the transforming growth factor beta (TGF-β) has a positive correlation with the mRNA expression of ZIP13 and contributes to the differentiation of beige adipocytes by regulating C/EBP-β protein levels [80,99]. ZIP13 deficiency accelerates adipocyte browning of the adipose tissue as C/EBP-β leads to adipogenesis and brown adipocyte differentiation [99]. As ZIP13 histamine residues are required for ZIP13-mediated zinc transport to suppress adipocyte browning, the inadequacy of this transporter causes an increase in C3H10T1/2 cells that can differentiate into beige or brown adipocytes when a brown adipogenic cocktail is administered [93,100]. It is also suggested that the interrupted zinc transportation from zinc stores [94], to the cytosol causes ER stress, leading to the development of Ehlers–Danlos syndrome, spondylodysplastic type 3 (EDSSPD3) [76,94]. The nuclear translocation of SMAD transcription factors in the BMP/TGF signaling pathway was reduced in ZIP13-knockout mice with phosphorylation status remaining unchanged, indicating that ZIP13 deficiency causes an impairment in BMP/TGF-β signaling pathway leading to abnormally shaped collagen-producing cell, shrinking cartilage, and poor chondrocyte differentiation, resulting in decreased collagen synthesis [76,93]. 

ZIP13 is also distributed in the intracellular vesicles [94]. Many enzymes requiring zinc are found in the secretory pathway, including Calnexin (Cnx) and calreticulin (Crt) in the ER, and glycosylphosphatidylinositol (GPI) phosphoethanolamine transferase and other zinc-secreted enzymes including matrix metalloproteases, alkaline phosphatases (ALP), and angiotensin-converting enzymes. ALPs can be an indicator of zinc deficiency in the ER and Golgi as they require zinc for their activity [94].

### 6.5. Analogies of ZIP7 and ZIP13

There are two sets of differentially expressed genes (DEGs) of both ZIP7 knockdown (KD) and ZIP13-KD in hMCS that overlap, indicating ZIP7 and ZIP13 share functional characteristics through the overexpression of shared genes [80].

ZIP7 and ZIP13 were found to have functional distinctiveness and similarities. ZIP7-KD and ZIP13-KD both upregulated ER stress-related genes, genes enriched in cysteine-type endopeptidase, wnt signaling pathway mechanisms, and blood coagulation. In addition, downregulated proliferation-related genes and genes enriched in glucocorticoid and hyp zip7 and zip13oxia responses as well as mRNA splicing processes. The downregulated genes were implicated in inflammatory and immunological responses indicating that both ZIP7-KD and ZIP13-KD are crucial for the immune response of hMSCs in either zinc-dependent and/or zinc-independent way.

Both ZIP7 and ZIP13 are equally necessary for collagen synthesis, as it was found that the knockout of ZIP7, with collagen promoter in control of Cre-Lox recombination, causes connective tissue disorders, corresponding to that of Zip13-knockout mice [76].

## 7. Zinc Deficiency-Related Skin Disorders

Approximately one-third of the world’s population suffers from zinc deficiency following a consequence of low zinc consumption, malabsorption, or increased loss. Low consumption of zinc is endemic to Southeast Asia, sub-Saharan Africa, rural Iran, Turkey, and Egypt [101,102]. Other zinc deficiency predisposing factors include a low-protein diet, a vegetarian diet, eating disorders such as anorexia nervosa or bulimia nervosa, parenteral nutrition, hookworm infection, AE, formula milk low in zinc, and other gastrointestinal and renal dysfunctions. Other zinc deficiency-related skin disorders include necrolytic migratory erythema [103,104], pellagra [105], and pressure ulcers [106].

In infants, zinc deficiency is attributed to either classic acrodermatitis enteropathica, maternal milk low on zinc, or premature infants with prolonged parenteral nutrition, all of which can be reversed upon zinc supplementation [107,108].

### 7.1. Acrodermatitis Enteropathica (AE)

Acrodermatitis is a zinc deficiency skin disease that is either hereditary or acquired [109]. It is attributed to zinc malabsorption in the duodenum or maldistribution of zinc to bodily tissues [110,111]. The latter can be diagnosed by identifying lower-than-normal ALP levels in blood serum [82]. Some studies suggested that the malabsorption of zinc is caused by a defect in the pancreatic zinc-binding ligands that transport zinc [112,113].

AE is characterized by the presence of cutaneous lesions that vary from mild to severe and are located around body openings and nails, with mild or severe diarrhea and alopecia [110,114]. Secondary infections due to immunosuppression are common and if AE is left untreated it can lead to death. Another form of AE showing zinc deficiency symptoms is observed in infants before weaning occurring as a result of reduced zinc secretion into the mother’s milk [115,116]. The identification of AE is performed by detecting low plasma zinc levels and it can be reversed with proper zinc supplementation [109]. 

### 7.2. Pathogenesis of AE

Zinc deficiency leads to elevating ATP and ADP levels and reducing adenosine levels in all cells due to extracellular adenine-nucleotide hydrolysis suppression in addition to causing a decrease in the activity of four major ectoenzymes (ENPP1, ENPP3, NT5E/CD73, and TNAP involved in the hydrolysis of extracellular ATP to adenosine through ADP and AMP). As a result, zinc affects extracellular adenine-nucleotide metabolism, and its deficiency slows both extracellular ATP clearance and adenosine production [117,118]. ZnT zinc transporters ZnT5 and ZnT6 heterodimers and ZnT7 homodimers may also increase vulnerability to zinc deficiencies as they are both required for TNAP and NT5E/CD73 activity [117,119]. It is also suggested that zinc deficiency causes a decrease in Langerhans cells that express ENTPD1/CD39 leading to severe acrodermatitis [120,121,122,123].

### 7.3. Spondylocheirodysplastic Ehlers–Danlos Syndrome (SCD-EDS)

EDS is an inherited disorder of connective tissue [124]. A new type of EDS was reported with slightly different features from that of the typical EDS [83]. The connective tissue disorders include hyperplastic skin, and articular hypermobility, while physical signs include wrinkled palms, short stature, tapered fingers, absence of periorbital tissue, and antimongoloid slant [125]. Similar clinical signs were observed in Zip13-KO mice, including impaired cartilage development, growth retardation, kyphosis, osteopenia, and craniofacial abnormalities with a reduction in corneal and dermal stromal collagen levels. SCD-EDS is attributed to a mutation in the ZIP13 protein of the LIV-1 subfamily [83].

### 7.4. Pathogenesis of SCD-EDS

Knocking out ZIP13 in mice revealed maturation defects in cells originating in the mesenchyme which led to retardation in connective tissue development [93]. Analysis revealed that ZIP13-KO cells demonstrate an impaired nuclear translocation of the transcription factor SMADs, responding to BMP and TGF-β necessary for connective tissue development [126,127]. An increase in zinc levels in the Golgi apparatus with a decrease in the nucleus was detected in Zip13-KO cells indicating a disruption in intracellular zinc homeostasis [83].

Therapeutic strategy for SCD-EDS can be achieved through restoring intracellular zinc homeostasis [94], and the removal of mutant ZIP13 protein via the ubiquitin-proteasome pathway [83]. VCP and HSP90 molecules are involved in the unfolding and transport of mutant ZIP13 to the proteasome. Inhibiting these molecules results in mutant ZIP13 protein accumulation within cells. The pathogenic ZIP13 mutants’ characteristics could be easily reversed by the proteasome inhibitors MG-132 and lactacystin [128]. These inhibitors are toxic, activating certain signaling pathways that can lead to cell death. Proteasomes take part in a variety of biological activities, such as cell proliferation, gene regulation, stress response, and apoptosis [129,130]. Therefore, proteasome-dependent degradation causes high cell toxicity [131,132,133]. For that, finding treatment remains challenging. Bortezomib is an example of a drug that was approved for human use but was later found to be causing cytotoxicity over the long term [134,135]. Proteasomes require protein-degradation folding molecules known as chaperones for the degradation of the cell membrane [136,137]. These chaperones can be targeted instead of proteasomes for eliminating pathogenic ZIP13 mutant protein in the treatment of SCD-EDS as they are not usually necessary for cell survival. Chaperone inhibitors, DBeQ and 17AAG were found to trigger the buildup of pathogenic ZIP13 mutant protein and restore zinc homeostasis within the cell [138]. Modification of chaperone inhibitors such as DBeQ and 17AAG to minimize cell toxicity can be an effective therapeutic strategy in the treatment of SCD-EDS [83].

## 8. Zinc in the Therapy of Skin Disorders

In dermatology, the topical application of zinc has been utilized widely in the treatment of various skin diseases of different etiologies as zinc possesses anti-bacterial and anti-inflammatory properties as well as offering photoprotection without causing adverse effects [5,6,139].

### 8.1. Acne Vulgaris

There are few theories that explain the pathogenesis of acne vulgaris [140,141,142,143]. In general, androgens and hyperkeratinization of the skin cause obstruction of sebaceous glands, thus, allowing the proliferation of *Propionibacterium acnes* bacteria. The latter causes inflammatory cells to gather at the site leading to metabolization of sebum’s triglyceride that forms free fatty acids and inflammatory mediators complex resulting in irritation.

Treatment protocols have been designed to tackle different stages in the pathogenesis of acne. These treatment methods can either be topical or systemic. Topical treatments include using wash gels and lotions or antibiotics, while systemic treatment includes the use of retinoids, hormonal mediators, or antibiotics. Both treatment methods have versatile side effects on the outer skin and internal organs, such as erythema, dryness, peeling, teratogenicity, high risks of embolism, and the development of antimicrobial resistance [144]. Through several clinical trials, it has been proved that zinc has the potential to reduce acne by inhibiting *P. acnes* proliferation, suppressing sebaceous gland activity, regulating DNA and RNA polymerases, and gene transcription [145,146,147]. It was found that combining topical zinc with erythromycin is superior to topical clindamycin or erythromycin alone in treating acne and is of equal effect with tetracycline [148]. It also showed an earlier onset of action compared to conventional treatment methods. Nels^®^, a zinc oxide-containing cream, was found to improve acne equally to benzoyl peroxide with fewer side effects [149]. Oral zinc or zinc plus oral vitamin A was found to outdo vitamin A alone [150,151].

### 8.2. Zinc in the Treatment of Other Skin Disorders 

Various skin disorders such as psoriasis and eczema can be successfully managed with zinc-containing compounds as shown in (Table 1).

## 9. Cosmeceutical Application of Zinc

Exposure to UV harmful sunrays of 320–400 nm (UVA) and 290–320 nm (UVB) can pose potential threats to the health and integrity of the skin’s connective tissue through initiating abnormalities such as melasma, variety of skin cancers, and photoaging. UVA was found to generate free radicals causing photoaging in human skin as well as promoting carcinogenesis and immunosuppression while UVB was found to be responsible for causing squamous cell carcinoma in animals [227,228,229,230].

As a UV blocking agent, FDA-approved ZnO has been widely employed in the formulation of inorganic (physical) sunscreens as a nonirritating, insoluble agent that can absorb, scatter, and reflect UV radiation of 290–380 nm responsible for triggering severe oxidative stress leading to DNA damage and apoptosis [231,232,233]. As a sunscreen component, microfine ZnO was found to outperform its microfine titanium dioxide alternative in providing better protection against long-wave UVA and in looking less white on the skin [234].

Initial sunscreen preparations lacked aesthetic appeal due to ZnO’s chalky white textural properties [235,236]. As the evolution in nanotechnology has emerged, ZnO particles were reduced to nanoparticles of less than 100 nm, which helped lessen the white chalky appearance. Generally, reduction in particle size has given rise to toxicity and oxidative stress concerns as well [237]. In the case of coated and noncoated ZnO-NP, it did not permeate the stratum corneum or cause local skin toxicity after an in vivo 5-day trial [238]. A large body of evidence supports the safe use of ZnO in sunscreen preparations and non-sunscreen preparations for the treatment of various skin disorders [6,139,238,239,240].

Since the human body has high levels of endogenous zinc, minimal transdermal absorption may not pose any adverse health effects as ZnO particles would dissociate into zinc and oxygen ions, both of which exist naturally in the human body [241]. The body’s homeostatic system controls the uptake, distribution, and excretion of zinc [242]. Respectively, oxygen absorption through the skin is safe and essential for life. The recommended ZnO concentration in sunscreen preparations should be no more than 25% according to the European Commission’s Scientific Committee on Consumer Safety [243].

Active ingredients reaching the viable layers of the skin used in combination with ZnO or Titanium Oxide in sunscreen preparations should be further investigated for their safe use as they can impact reproduction, development, or carcinogenesis [244].

As a waste material, a study evaluating the safety of anthropogenic inorganic sunscreen filters in seawater waste on coral reefs revealed that uncoated ZnO nanoparticles can cause severe, rapid bleaching of *Acropora* spp. [245]. 

## 10. Conclusions

Zinc is a trace element that plays a major role in maintaining healthy status since early infancy until elderliness. Subsequently, changes in required zinc levels can result in different dermatological disorders that can mostly be reversed with adequate zinc supplementation, while the involvement of abnormally functioning zinc transporters can limit the efficacy of zinc supplementation. Zinc-containing compounds remain a favorable therapeutic option for various dermatological disorders due to its lack of serious side effects upon topical use. Zinc and zinc transporters have been extensively investigated in the past few decades, but a large body of evidence is still missing such as a clear understanding of precise subcellular zinc transportation following cellular stimuli, each zinc transporter structure and their efflux and influx mechanisms, or the identification of the specific components mediated by zinc and their functions in protein networking. In the formulation of sunscreens, ZnO’s UV rays scattering and reflecting properties allowed for its extensive and safe use on daily basis, but the safety of chemical UV-filters used in combination with ZnO require further investigations.

## Figures and Tables

**Table 1:** Therapeutic applications of zinc in the treatment of various skin disorders.

Disorder	Etiology	Treatment	References
Acne conglobata	Propionibacterium acnes	Successfully treated with a high dose of oral zinc sulphate.	[152]
Acne vulgaris	Propionibacterium acnes	Clindamycin or erythromycin in combination with zinc acetate or octoate was found to boost therapy efficacy.	[145,146,150,153,154,155,156,157,158,159,160,161,162]
		Papular and pustular acne can be cured with oral zinc sulphate.	
		Oral zinc gluconate is effective in the management of inflammatory acne.	
		Antioxidants combined with methionine-bound zinc complex was successful in treating mild to moderate conditions.	
		The alternative route of treatment can be zinc alone or in combination with nicotinamide.	
Actinic keratosis	UV exposure	Topical 25% zinc sulphate resulted in the disappearance of the lesions.	[163]
Alopecia areata	autoimmune disorder	Oral zinc supplementation showed a noticeable clinical response.	[164,165]
Athlete’s foot	Trichophyton rubrum	20% zinc-undecylenate-containing powder was found to be effective in reducing erythema, scaling, and itching.	[166,167]
Androgenetic alopecia	androgens, genetic predisposition	Significant hair growth was observed with topical zinc pyrithione 1% solution.	[168,169]
Behcet’s disease	autoimmune disorder	Behcet’s disease was treated with oral zinc sulphate.	[170,171]
Bromhidrosis	*Corynebacterium* sp.	Topical zinc salt such as sulphate and zinc oxide were found to be successful in the management of the condition.	[172,173]
Bromodosis	Sweat buildup leading to bacterial or fungal growth	A topical 15% zinc sulphate solution was found to eliminate foot odor.	[174]
Cutaneous leishmaniasis	Leishmania	Intralesional 2% zinc sulphate with meglumine and oral zinc sulphate was found to be effective in the management of cutaneous leishmaniasis.	[175,176,177]
Dissecting cellulitis of the scalp	Unknown	Complete cure with oral zinc sulphate.	[152,178,179]
Eczema	Immune system overactivity	Textiles treated with zinc oxide can be useful in the management of atopic dermatitis.	[180,181,182]
		For diaper dermatitis, zinc oxide paste was found to be effective in soothing and preventing skin rash.	
		For hand eczema, a cream containing zinc sulphate (2.5%) combined with clobetasol (0.05%) has improved the condition.	
Erosive pustular dermatosis of the scalp	Unknown	Treated with oral zinc sulphate.	[183]
Herpes genitalis	Herpes simplex virus type 2	Zinc acetate gel was effective in the prevention of sexual transmission of HSV-2 and HIV.	[184,185]
		Higher concentrations of zinc sulphate were found more effective in the treatment, and prevention of relapse.	
Herpes simplex	Herpes simplex virus type 1	Polyethylene glycol-coated zinc oxide nanoparticles demonstrated antiviral potency against HSV-1	[186,187]
		Zinc gluconate and zinc lactate was found to effectively inactivate HSV-1 clinical isolates.	
Hidradenitis suppurativa	Unknown	The disorder can be managed with oral zinc gluconate alone or in combination with topical triclosan.	[188,189]
Jock itch	Trichophyton rubrum Trichophyton mentagrophytes	A cream formulated with 20% zinc undecylenate has effectively cleared the skin.	[166,190]
Leprosy	Mycobacterium leprae	Combining oral zinc with dapsone was found to enhance the therapy’s effectiveness through bacterial clearance and rapid conversion of lepromin.	[191,192,193]
		Topical application of phenytoin sodium zinc oxide paste showed a significant clearance of the bacterial load of trophic ulcers.	
Melasma	UV exposure	Topical 10% zinc sulphate resulted in a significant decrease in MASI* scores.	[194,195,196,197,198,199]
		Zinc oxide in sunscreen formulations is used in the management of melasma owing to its photoprotection properties.	
Necrolytic acral erythema	Associated with hepatitis C	The condition was treated with oral zinc supplementation.	[200,201,202]
Necrolytic migratory erythema	Associated with pancreatic glucagonoma	Oral zinc sulfate has been shown to improve the condition.	[203]
Oral aphthous stomatitis	Unknown	Oral zinc sulphate lowered the risk of relapse in recurrent aphthae and provided both curative and preventative effects.	[204,205,206,207,208]
		Zinc sulphate-containing mouth rinse decreased the frequency of recurring ulcers.	
Oral lichen planus	Unknown	0.2% zinc mouthwash in combination with fluocinolone helped diminish irritability, pain, and lesion surface area.	[209,210]
		Administration of oral zinc acetate showed favorable clinical improvement.	
Pityriasis versicolor	Malassezia	Zinc pyrithione 1% in shampoo formulations was found effective in the treatment of pityriasis versicolor.	[211,212,213,214]
		Topical 15% zinc sulphate was effective in the treatment of pityriasis versicolor.	
Psoriasis	Unknown	Topical 0.25% zinc pyrithione was found effective for localized plaque psoriasis.	[215]
Psoriatic arthritis	Unknown	Psoriatic arthritis can be effectively treated with oral zinc sulphate.	[148,216]
Seborrheic dermatitis	Malassezia	Zinc pyrithione 1% in a shampoo formulation is a therapeutic choice for reducing inflammations and scaling.	[215]
Ulcers	Poor blood flow	Topical zinc oxide formulations have been used in the treatment of arterial and venous leg ulcers, pressure ulcers, and diabetic foot ulcers.	[6,217,218,219,220]
		Zinc iontophoresis was demonstrated to be beneficial in the treatment of ischemic skin ulcers.	
Vitiligo	Melanocyte decrement in relation to genetic and non-genetic factors	Oral zinc sulphate in combination with topical corticosteroids showed a higher response rate than Topical corticosteroids alone in the treatment of vitiligo.	[221]
Warts	Human papillomavirus	Topical 10% zinc sulfate was found effective for the treatment of plane warts	[222,223,224,225,226]
		Oral zinc sulfate can be used in the treatment of different types of warts.	
		Topical 20% zinc oxide is considered an effective and safe therapeutic method.	
		Zinc acetate coformulated in a carrageenan gel demonstrated anti-HIV and anti-human papillomavirus activity.	

MASI*: melasma area and severity index.

## Data Availability

Not applicable.
